# Adverse Event Circumstances and the Case of Drug Interactions

**DOI:** 10.3390/healthcare7010045

**Published:** 2019-03-19

**Authors:** Theodoros G. Soldatos, David B. Jackson

**Affiliations:** Molecular Health GmbH, Kurfuersten Anlage 21, 69115 Heidelberg, Germany; soldatos@molecularhealth.com

**Keywords:** adverse events, drug-drug interactions, health informatics, outcome analytics, molecular mechanism based safety assessment, real world data, public health

## Abstract

Adverse events are a common and for the most part unavoidable consequence of therapeutic intervention. Nevertheless, available tomes of such data now provide us with an invaluable opportunity to study the relationship between human phenotype and drug-induced protein perturbations within a patient system. Deciphering the molecular basis of such adverse responses is not only paramount to the development of safer drugs but also presents a unique opportunity to dissect disease systems in search of novel response biomarkers, drug targets, and efficacious combination therapies. Inspired by the potential applications of this approach, we first examined adverse event circumstances reported in FAERS and then performed a molecular level interrogation of cancer patient adverse events to investigate the prevalence of drug-drug interactions in the context of patient responses. We discuss avoidable and/or preventable cases and how molecular analytics can help optimize therapeutic use of co-medications. While up to one out of three adverse events in this dataset might be explicable by iatrogenic, patient, and product/device related factors, almost half of the patients in FAERS received multiple drugs and one in four may have experienced effects attributable to drug interactions.

## 1. Introduction

The identification of novel adverse events (AEs) is critical to the protection of patient well-being and the healthcare system that supports them. From the induction of avoidable and sometimes fatal side effects to the billions of dollars in associated medical costs, AEs remain a critical issue for all stakeholders in the healthcare system [1,2]. Different studies report that more than six percent of acute hospital admissions are caused by serious adverse reactions to medicines in the US and the EU [3,4,5,6], while preventable medical errors may be accountable for as much as the third leading cause of death in the US [7]. One particular category of such preventable patient safety issues is drug-drug interactions (DDIs) [8,9,10].

We considered the landscape of circumstances pertaining to adverse event cases captured within the US Food and Drug Administration (FDA) Adverse Event Reporting System (FAERS). FAERS is a database that contains information on AE and medication error reports submitted to the FDA, designed to support its post-marketing safety surveillance program for drug and therapeutic biologic products [11]. AEs and medication errors are coded in terms from the Medical Dictionary for Regulatory Activities (MedDRA) terminology. However, one key consideration that limits the usefulness of AE data is the lack of direct molecular characterization, making it difficult to generate hypotheses regarding the causality of an AE [12]. To enable both scientists and statisticians to make maximal use of AE data, patient records must be linked to system level views on drug mode of action [12]. Such an approach might be able to also help systematically prevent critical medication errors, for example, by the timely identification of predictable DDIs. Focusing on cancer patients, where drug interactions from polypharmacy is a potential concern, we employ this methodology and demonstrate that molecular profiling of individual patient prescriptions is indeed one important parameter that can help optimize therapeutic use of drugs in the form of detecting, preventing, and replacing problematic combinations.

## 2. Materials and Methods

**Figure 1:**
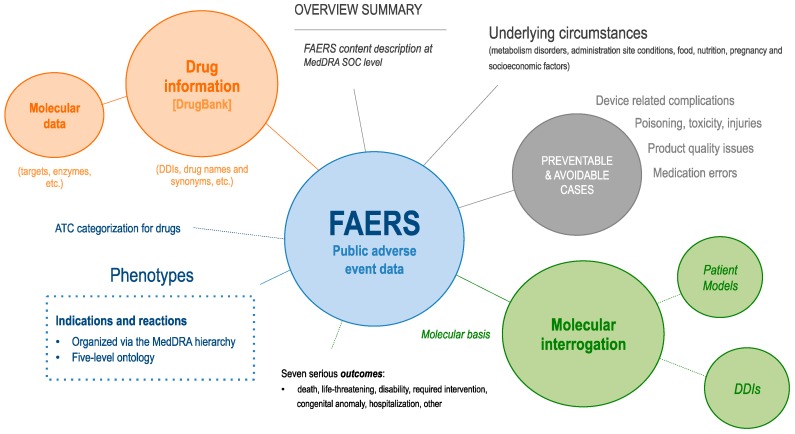
Synopsis of our Food and Drug Administration (FDA) Adverse Event Reporting System (FAERS) content study. First, we enriched FAERS data with DrugBank, Anatomical Therapeutic Chemical (ATC), and Medical Dictionary for Regulatory Activities (MedDRA) information. We then review the observed indication and reaction occurrence at different MedDRA terminology levels and finally examine molecular dependencies at the patient level and examine drug interaction rates.

We firstly reviewed the landscape of AEs in FAERS and then analyzed the underlying circumstances to decipher their potential molecular causation. Figure 1 summarizes our study.

### 2.1. Datasets

We integrated clinical data for about 6.8 million patients from the FAERS database (2000–2016), with molecular knowledge from the DrugBank [13] database (drug names and synonyms, known/label DDIs, drug-targets, and drug-metabolizing enzymes) [12]. This FAERS dataset held about 8.2 million drug safety reports containing information about each patient’s treatments, disease indications, and side effects (termed reactions) coded using the MedDRA ontology [14]. To compensate for the ambiguity introduced by the use of non-standardized drug names [12,15], free text treatment descriptions mentioned in FAERS safety reports were matched to associated DrugBank records [12]. Further levels of integration were achieved by using the Anatomical Therapeutic Chemical (ATC) ontology from the WHO (v2016) [16] and the full hierarchical structure of MedDRA (v12.1).

### 2.2. Experiments

We summarized FAERS content at the ‘System Organ Class’ (SOC) level (i.e., highest category) from the MedDRA hierarchy and discuss top reported indications and reactions, as well as food, nutrition, pregnancy, and socioeconomic conditions. Then, we searched FAERS for possible preventable and/or avoidable incidences and characterize individual patient AEs at the molecular level.

#### 2.2.1. Preventable and Avoidable Errors

To identify the number of AEs that may include preventable and avoidable errors, we extracted High Level Terms (HLTs) from MedDRA (i.e., MedDRA level 3 category terms) linked to more than 1000 AEs as reactions (see Supplementary Data). We then manually selected the top (most frequently mentioned) terms that did not refer to physiological states (disease, conditions, side effects) or to circumstances examined previously in this work. More specifically, we considered the following MedDRA HLTs: ‘Therapeutic and nontherapeutic responses’; ‘Non-site specific injuries NEC’; ’Therapeutic procedures NEC’; ‘Substance-related disorders’; ‘Allergic conditions NEC’; ‘Suicidal and self-injurious behavior’; ‘Allergies to foods, food additives, drugs and other chemicals’; ‘Poisoning and toxicity’; ‘Interactions’; ‘Withdrawal and rebound effects’; ‘Drug and chemical abuse’; ‘Tobacco use’; ‘Maladministration’; ‘Overdoses’; ‘Non-site specific procedural complications’; ‘Medication errors due to accidental exposures’; ‘Product quality issues NEC’; ‘Device related complications’; ‘Device malfunction events’; ‘Device component findings’; ‘Product physical issues’; ‘Product packaging issues’; ‘Product contamination and sterility issues’; and ‘Product label issues’.

#### 2.2.2. Individual Patient Molecular Models for Optimizing Therapeutic Use of Combinatorial Treatment Regiments

For this analysis, we considered hospitalization, death, and life-threatening cases by examining those AEs that reported the respective outcomes (namely, ‘Hospitalization—Initial or Prolonged’, ‘Death’, and ‘Life-Threatening’). Cancer patients were defined as those for whom AEs reported indications linked to the MedDRA SOC (i.e., level 1 category) termed ‘Neoplasms benign, malignant and unspecified (incl. cysts and polyps)’. Similarly, for the lung cancer cohort, MedDRA HLT ‘Non-small cell neoplasms malignant of the respiratory tract cell type specified’ (level 3 category term) was used. Similarly, breast cancer cases were defined to refer to patients whose reported indications linked to the MedDRA High Level Group Term (HLGT) ‘Breast neoplasms malignant and unspecified (incl. nipple)’ (level 2 category term). For prostate cancer cases, the indications linked to the MedDRA HLT ‘Prostatic neoplasms malignant’ (level 3 term), whereas for the endometrial neoplasm cohort patients reported indications linked to the MedDRA HLT ‘Endometrial neoplasms malignant’ (level 3 term). Lastly, the skin cancer cohort was defined to be those patients that had cases reporting indications linked to MedDRA HLGT, ‘Skin neoplasms malignant and unspecified’ (level 2 term) and anti-cancer drugs that linked to the top-level term, ‘Antineoplastic and Immunomodulating Agents’ from ATC (code ‘L’).

## 3. Results

We examined 6,791,370 AE cases from FAERS. The dataset contained 6,791,348 cases (99.9% FAERS) with reports linked to free text medication descriptions, of which 6,601,199 (97.2% FAERS) were annotated with drugs and therapeutic products or supplements. In 3,133,801 AEs (46.1% FAERS) multiple drugs were mentioned across their case-reports, indicating possible polypharmacy. Indication and reaction information was available for 5,791,090 (85.3% FAERS) and 6,791,321 (99.9% FAERS) AE cases, respectively. Table 1 summarizes indication and reaction occurrence in MedDRA SOC (‘System Organ Classes’, highest level) terms. Only 38,127 and 3,858 cases reported reactions that explicitly mentioned the terms ‘Adverse event’ or ‘Adverse reaction’ correspondingly, and 40,246 cases reported ‘Adverse effect absent’.

### 3.1. FAERS Overview

While the general MedDRA SOC categories ‘Investigations’ and ‘Surgical and medical procedures’ reflect therapeutic processes and tests applied, the occurrence of most MedDRA SOC categories was higher when reported as reaction than as indication. Exceptions included cancer, endocrine, and immune system disorders. Most reported indications included musculoskeletal, connective tissue, immune, and nervous system disorders, followed by metabolism-related and cancer cases. Most frequent reactions included nervous, gastrointestinal, cardiac, respiratory, skin, and psychiatric disorders.

The high occurrence of classes such as ‘Injury, poisoning and procedural complications’ and ‘Infections and infestations’ as reactions may provide an explanation for some AEs. However, looking at these data alone cannot provide a causative explanation. We therefore decided to investigate further categories that may describe AE occurrence irrespective of intrinsic physiological, disease, or medical implications.

#### 3.1.1. How May AEs Be Explained?

We examined highly reported indication and reaction categories, and categories that referred to circumstances, other than physiological states (diseases, side-effects). More specifically, we examined the two most frequent reaction categories mentioned in FAERS (i.e., ‘General disorders and administration site conditions’ and ‘Injury, poisoning and procedural complications’ that linked to 39.94% and 21.2% FAERS, respectively; Table 1), and then considered the occurrence of food, nutrition, and pregnancy related incidences (Table S1). We also considered the impact of other socioeconomic circumstances (Table S2).

We noticed that administrative site reactions, (not otherwise classified) injuries, and therapeutic and non-therapeutic effects were reported in several cases (4.8%, 5.28%, and 10% FAERS, respectively); Table S1. Regarding metabolism, the main cases included appetite and general nutritional disorders (2.3% FAERS, as reaction), bone, calcium, magnesium, and phosphorus issues (2.16% FAERS, as indication), as well as electrolyte and fluid balance conditions (3.85% FAERS, as reaction); Table S1. Glucose metabolism disorders (including diabetes mellitus) were mentioned in several AEs, both as indications and reactions (4.89% and 1.53% FAERS, respectively); Table S1. Specific food intolerance syndromes and vitamin related disorders were, however, uncommon (<1% FAERS); Table S1.

Regarding other circumstances, we found that pregnancy issues were mainly reported as reactions (1.74% FAERS), with key complications including abortion and stillbirth, fetal, neonatal, and perinatal conditions; Table S1. Specific socioeconomic aspects were mentioned in a few AEs only (1.53% FAERS, as reactions); Table 1. Age-related factors were noted more as indications, whereas legal, family, housing, and legal/crime issues were mainly reported as reactions; Table S2. Importantly, at least 1.28% of FAERS reports explicitly referred to lifestyle issues; Table S2.

Yet, these results cannot help derive conclusive hypotheses on AE causes or their impact over AEs. First, the observed occurrences are based on AE reports only and are thus biased since some aspects may be over/under-represented, especially in the absence of appropriate control/reference data. Thus, independence from disease, physiological, or medicine-affected conditions cannot be verified, as some of the so far examined categories may be influenced directly by previous, current, and ongoing therapies or co-morbidities.

#### 3.1.2. Preventable and Avoidable Errors

While these factors make it difficult to understand the landscape pertaining AEs, several incidences suggest that some AEs may be avoidable or preventable through changes in therapeutic regimen or more appropriate handling and care (e.g., lifestyle, injuries, etc.). Next, we examined separately device, product, injury, and poisoning and procedural complications more in detail.

We extracted MedDRA HLTs (i.e., level 3 terms) linked to more than 1000 AEs as reactions (see Supplementary Data) and manually selected the top 20 that did not refer to physiological states (disease, conditions, side effects) or to circumstances examined earlier in this work. Table S3 summarizes those categories and lists respective reactions from each class mentioned in at least 500 AEs each.

Overall, we found several incidences that could be prevented or avoided. Even though in some cases it cannot be clear if an event was an error (e.g., drug toxicity, over/under-dose) or to which circumstances (patient, clinical, genomic, practical factors, etc.) it might be attributable (e.g., off-label use, drug ineffective, drug resistance, over/under-dose, drug withdrawal, etc.). Therefore, we roughly grouped those reactions within the following categories:

- Patient factors: 3.43% FAERS reported accidents, 2.62% FAERS reported off-label use or self-medication, 1.99% FAERS reported substance related disorders (drug abuse, alcoholism); 1.89% FAERS reported multiple allergic conditions and 1.25% allergies to foods, food additives, drugs, and other chemicals (e.g., drug hypersensitivity); 0.75% FAERS reported drug withdrawal and rebound effects; and 0.14% FAERS reported accidental exposures (e.g., intake by child, via breast milk).
- Iatrogenic, therapeutic, and procedural: 4.76% FAERS reported maladministration errors (wrong technique or drug, inappropriate site, incorrect duration or dose); 1.48% FAERS reported different types of overdoses (intentional, accidental, due to multiple drugs); 1.12% FAERS reported different forms of poisoning or toxicity; 1.03% FAERS reported procedural complications or iatrogenic injury; 0.98% reported interactions (drug-drug, alcohol); 0.87% FAERS reported medication errors (prescribing, dispensing, interception); and 0.05% FAERS reported product label issues (confusion, lot number, expiration date).
- Device and product issues: 1.91% FAERS reported product quality issues (storage, formulation); 0.88% FAERS reported device related complications (infection, misuse, accident); 0.59% FAERS reported device malfunctions (dislocation, occlusion, delivery system); 0.28% FAERS reported device component findings (breakage, failure, leakage); 0.14%; and 0.09% FAERS reported product physical and packaging issues (abnormal color, size, odor, solubility, container, quantity).

Moreover, 1.62% FAERS reported suicidal and self-injurious behaviors and 8.53% FAERS cases reported reactions related to therapeutic responses (none, unexpected, delayed, increased), drug tolerance, resistance, and effectiveness. Put together, those categories alone (examined in this section) accounted for up to 1,954,285 AEs (28.78% FAERS).

### 3.2. Molecular Level Interrogation at the Patient Level

Next, we wondered whether other ways might exist that would allow not only dissecting AE information but also helping potentially interpret observed effects. In specific, we integrated drug-induced phenotypes (indications, reactions, outcomes) from FAERS with the molecular world of drugs and targets [12] to investigate how molecular interrogation of AE reports may permit deeper understanding of AE circumstances. Table 2 summarizes key aspects enabled by this approach. Importantly, physiological effects can be deciphered by exploring the interactions of drugs and other molecular components [12].

Here, we apply this methodology to demonstrate that molecular analysis of patient prescriptions can also help generate insights regarding drug interaction safety and therapeutic use of drugs.

#### 3.2.1. Polypharmacy in Cancer

**Figure 2:**
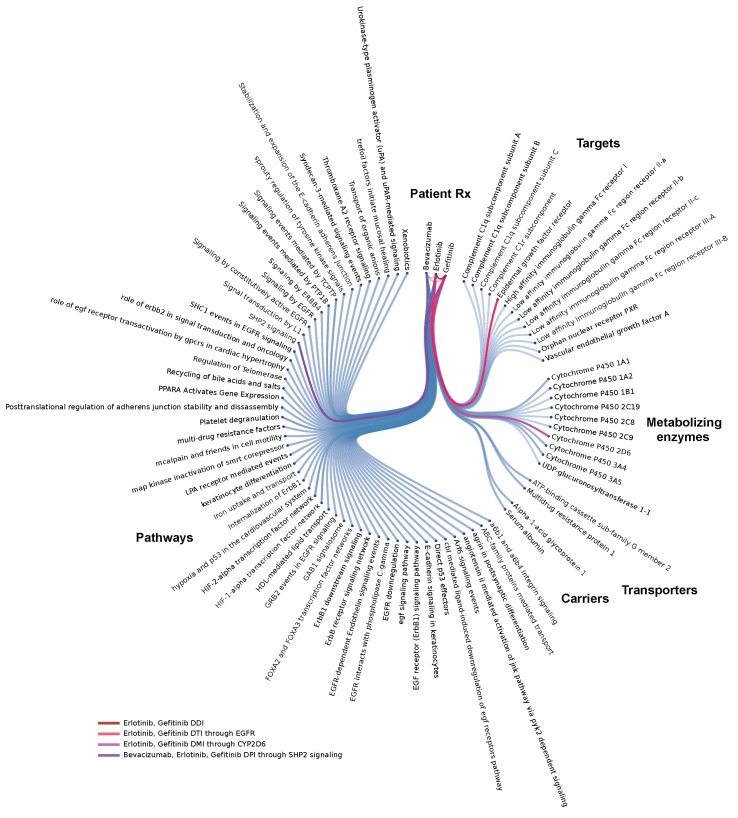
Systems-level analysis of drug-interactions: Example of an individual patient-specific case-model via a Molecular Dependency Graph: this systems pharmacology molecular model highlights co-occurrence of label-based, target-, metabolizing enzyme-, and pathway-level interactions in a single patient. This lung cancer patient’s model (FAERS #6703852) involves co-medication of erlotinib with gefitinib, a clinically problematic combination, probably due to overloading of the drug-binding site on the Epidermal Growth Factor Receptor (EGFR) within the target tissue.

In almost half of FAERS cases (46.1%) patients may have received multiple drugs simultaneously or during the course of their therapy. We therefore decided to perform molecular analysis of prescriptions on a single patient level: forming molecular models of individual patient cases can help identify combinations that could be clinically problematic (e.g., due to overloading of the drug-binding site of a common target or due to competitive inhibition at the level of metabolizing enzymes) or generate a hypothesis about clinical response and the influence of co-medications. Such patient specific disease model views can be generated for all AEs in FAERS (Figure 2).

The clinical response to antineoplastic interventions is influenced by tumor and patient intrinsic factors that largely derive from genomic diversity. Non-cancer co-medications are an extrinsic factor that can act analogously by perturbing the function of genes involved in mediating the therapeutic effect of an anti-cancer drug. By interfering at the molecular level, co-medications have the potential to alter cancer drug responsiveness and, in particular, affect side effect and outcome profiles. This is a critical consideration as many cancer patients are poly-medicated. With the goal of analyzing the potential contribution of such factors, we characterized the prevalence of co-medication interactions and of adverse events or negative clinical outcomes in cancer patients. Importantly, one 1 of 10 AEs in FAERS referred to a cancer patient.

#### 3.2.2. Drug interaction Complications

We examined AE case reports for known DDIs and for potential interactions between drugs that can occur at the level of targets (DTIs) or metabolizing enzymes (DMIs). We also examined the prevalence of three classes of negative clinical outcomes (death, life threatening, hospitalization) in the context of five cancer indications. We also asked what proportion of each phenotype’s cases occurs in patients with potential interactions (DXIs) at any of the examined levels of the patient system (i.e., any of DDIs, DTIs, or DMIs).

**Figure 3:**
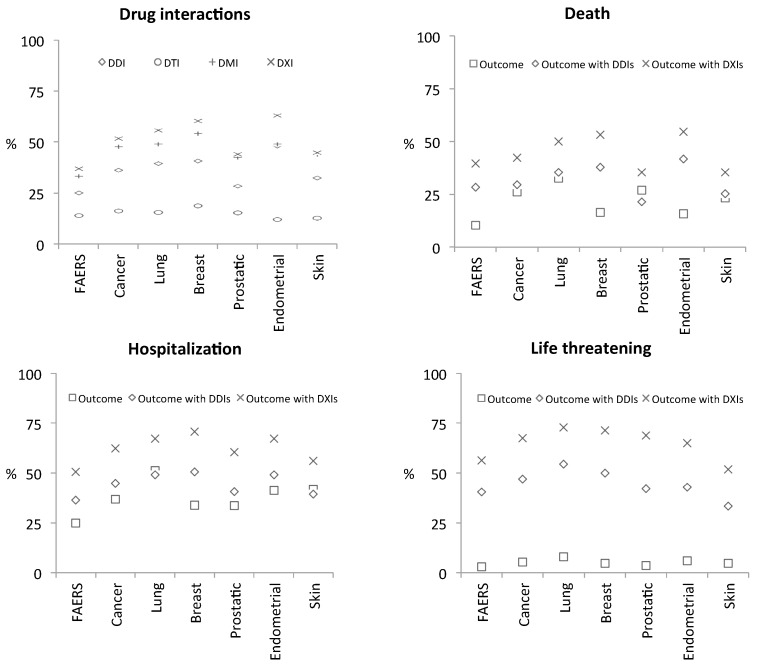
Synopsis of system-level drug-interaction rates and of selected clinical phenotypes detected in FAERS case-reports. Top-left graph (Drug interactions): each FAERS case-report was analyzed for potential drug-interactions (DXIs) as contraindicated in drug-labels (i.e., known/label DDIs) and with respect to interactions that can occur at the level of targets (DTIs) and metabolizing-enzymes (DMIs). Results were then grouped with respect to five cancer-indications (lung, breast, prostate, endometrial, and skin neoplasms). Percentages (%) reflect how frequently interactions occur in the different cohorts based on the number of cases that a certain drug-interaction type (namely, DDIs, DTIs, DMIs, or any of those, i.e., DXIs) was detected in that context. Remaining graphs (Outcomes ‘Death’, ‘Hospitalization’, and ‘Life threatening’): to assess the effect of co-medications on clinical response we asked what proportion (%) of each phenotype’s cases, per cohort, occur in patients with DDIs (rhombus symbol) and DXIs (x symbol)—we observe that DXI prevalence among each phenotype remains high irrespective of the disease-indication (x-axis), or the actual rate of that phenotype within each cohort (square symbols; proportion of phenotype within patient-cases of each cohort).

A synopsis of the results is provided in Figure 3: known DDIs were identified in 23.96% of FAERS cases, whereas in the skin cancer cohort, for example, this number rises to 31.16%, and up to 35.25% among all cancer patients. Overall, system-level DTI and DMI interactions are also common in cancer patients and serious outcomes occur to a large extent within DXI cases (Supplementary Data). For example, while 41.82% and 23.26% skin cancer patient cases reported hospitalization and death as outcomes respectively, 56.32% and 35.44% of those occurred in patients with DXIs indicating use of multiple medications that may affect each other. This emphasizes that co-medications are a critical factor that can potentially impact patient response. For example, only 13.6% of the medications reported in skin cancer cases were antineoplastic interventions, again emphasizing extensive poly-medication.

Even in absence of direct effects on anti-cancer drug activity, co-medications may interact with each other to cause AEs that can exacerbate or cause other patient co-morbidities. These clinical scenarios can potentially mask the true therapeutic effects of an otherwise effective anti-cancer medication and thereby negatively affect patient response to treatment. In other cases, such AEs may be falsely attributed to the anti-cancer medication.

## 4. Discussion

With this work, we attempted to investigate the landscape underlying the occurrence of AEs as reflected by public FAERS data. However, one cannot be certain about the actual circumstances underlying those cases without associated clinical narratives or control data. Moreover, studying side effects is complicated, as a combination of molecular interactions can affect the pharmacodynamic properties of drugs in different ways. Nevertheless, it is possible to capitalize on rich sources of clinical reports, such as FAERS, by combining them with additional data; for example this enables sufficient data to exist that allow studying the relationship between molecular mechanisms and drug-induced phenotypes so as to learn about—and even predict—AEs in patients [12].

In the context of this work, we demonstrated that individual patient molecular models could help generate hypotheses for optimizing therapeutic use of combinatorial treatment regiments. AE data provides an extensive body of the human phenotypes that have been observed in the context of real-world combinatorial treatments. By combining this data with molecular targets, we enable the analysis of the mechanisms that may underlie each AE. Such insights may help in improving patient treatment and avoid erroneous prescriptions or possibly dangerous combinations of therapies [12,17]. Preventing such iatrogenic AEs is one only example of potent therapeutic, research, and clinical applications of this methodology.

In this work, we found that one in three AEs may be explicable by avoidable and preventable cases (28.78% FAERS). Our analysis revealed that the potential for improvement may be even greater, since almost half of the events in FAERS may have included some sort of polypharmacy related effect (46.1%). For example, cancer patients—who reflect one tenth of FAERS cases—typically are treated with multiple therapies, when known DDIs were identified in 23.96% of FAERS cases. Importantly, to a large extent serious outcomes and reactions were found to occur within such drug interaction cases. Even though this observation may somewhat reflect that ‘sicker’ patients may receive more drugs and suffer worse outcomes, it also does invite for the development of more computational strategies that search among reported AEs for significant drug-induced phenotypic effects (e.g., increased side effects or positive survival effects). In principle, a set of systems-based analytical approaches should be developed to systematically generate clinical hypotheses and mine clinical reports for evidence of efficacious target and/or drug combinations, either in the form of target and/or drug combinations with increased side effect occurrence, or in the form of target combinations that appear to alter (induce or attenuate) certain drug side effects [18,19].

While drug safety is critical to the protection of patient wellbeing, development of digital applications based on the molecular analysis of clinical reports can offer rich benefits to the healthcare system as a whole: industry, clinics, education, and research [12,17]. This is especially important within the evolution of context-aware applications for disease management and the current development efforts focused on providing efficient infrastructures in computational treatment decision support. In addition, empowering physicians and pharmacists with the knowledge they require to confidently select the most appropriate therapies with maximum cost-effectiveness and clinical success is crucial in personalized medicine.

Integrating broader definitions of DDI information in the healthcare system is one such example, helping avoid this type of potential medication errors. However, DDI knowledgebases need to take into account more patient-specific information [20]. While several databases exist that summarize known label and peer-reviewed literature information [21,22,23], there is significant scope for new innovations to systematically incorporate and coordinate full interaction alert systems (including information about allergies, specific disease circumstances, laboratory metrics, dosage, time, and other parameters) across the broad healthcare system spectrum (personalized health apps, patient bedside, pharmacy, clinical practice, etc.).

The ability to identify potential or predicted DDIs becomes more important, when considering that it is not feasible to run interaction assays for every possible combination (or mechanism) as the number of approved and over-the-counter drugs grows larger. In this study we focused on cancer AEs because polypharmacy is common among those patients. However, onco therapy duration is usually limited in time and therapeutic options are typically linked to specific guidelines, meaning that co-medications for other ailments need to be analyzed and changed if necessary in order to avoid some of the negative outcomes reported here. In the future, we plan to explore the broader utility of our approach in additional clinical contexts, therapies, and conditions, such as cardiovascular diseases or metabolic syndrome. We also aim to automate our DDI characterization process and compare with results from updated FAERS and DDI datasets, as well as report on content from additional AE resources such as EudraVigilance [24] or VigiBase [25].

## 5. Conclusions

Overall, mechanism-based safety assessment approaches [26,27,28] are important for the development of strategies to improve healthcare [29], especially when considering the magnitude of downstream medical costs related to poor patient outcomes and drug-related mistakes [30,31] or the extent of iatrogenic medical errors [7]. Such sources of preventable drug-related harm include serious dose related reactions and DDI-induced AEs. Our molecular modeling of patient prescriptions highlighted the prevalence of DDIs in the context of patient responses showing that molecular analytics for optimizing therapeutic use of co-medications requires further attention.

Our drug interaction analysis results also highlight that more must be done to protect the therapeutic fidelity both of anti-cancer drugs and other patient co-medications. Target profiling of individual patient prescriptions is one important parameter that can help optimize therapeutic use in the form of detecting, preventing, and replacing problematic combinations.

## Figures and Tables

**Table 1:** FAERS indications and reactions summarized at MedDRA System Organ Class (SOC, level 1) terms. Indication and reaction information from FAERS is not straightforward to interpret, as many cases do not have indications as well as in many cases the reported reactions may refer to similar conditions or may reflect the patient’s disease. * AEs: adverse events.

MedDRA SOC Name	Indication AEs *	%Total AEs	Reaction AEs	%Total AEs
Blood and lymphatic system disorders	350,726	5.16	382,653	5.63
Cardiac disorders	208,679	3.07	1,075,568	15.84
Congenital, familial and genetic disorders	56,330	0.83	50,759	0.75
Ear and labyrinth disorders	8,733	0.13	91,631	1.35
Endocrine disorders	448,743	6.61	218,981	3.22
Eye disorders	69,279	1.02	315,958	4.65
Gastrointestinal disorders	452,855	6.67	1,349,645	19.87
General disorders and administration site conditions	247,082	3.64	2,712,307	39.94
Hepatobiliary disorders	126,044	1.86	203,980	3.00
Immune system disorders	799,829	11.78	477,511	7.03
Infections and infestations	350,316	5.16	770,049	11.34
Injury, poisoning and procedural complications	58,190	0.86	1,439,977	21.2
Investigations	242,129	3.57	954,804	14.06
Metabolism and nutrition disorders	644,521	9.49	632,797	9.32
Musculoskeletal and connective tissue disorders	922,255	13.58	930,144	13.7
Neoplasms benign, malignant and unspecified (incl. cysts and polyps)	671,890	9.89	348,083	5.13
Nervous system disorders	784,039	11.54	1,745,201	25.70
Pregnancy, puerperium and perinatal conditions	17,004	0.25	118,320	1.74
Psychiatric disorders	543,341	8.00	1,054,377	15.53
Renal and urinary disorders	179,233	2.64	433,585	6.38
Reproductive system and breast disorders	246,009	3.62	296,533	4.37
Respiratory, thoracic and mediastinal disorders	449,304	6.62	1,077,425	15.86
Skin and subcutaneous tissue disorders	375,556	5.53	994,511	14.64
Social circumstances	16,268	0.24	103,877	1.53
Surgical and medical procedures	968,612	14.26	325,664	4.80
Vascular disorders	500,778	7.37	1,410,722	20.77

**Table 2:** Benefits of molecularly exploring clinical reports. Integrating AEs together with drug and molecular data can potentially facilitate a number of valuable utilities: molecular components and interactions can reveal the mechanisms of action underlying AEs and explore information from the perspective of potentially causative molecular mechanisms. Exploration of underlying molecular mechanisms allows potential interpretation of the observed physiological effects, investigating the combined effect of combo-therapies and discover potentially unknown mechanisms responsible for observed fatal, poor, or unexpected outcomes [12].

Key Utilities Enabled by the Molecular Dissection of Patient Clinical Data
Biosystem level analysis of drug mode of action and safety. Clinical reports can be linked to human disease models. Molecular analytics to expedite the identification and understanding of safety problems. Comparative analysis of safety profiles between drugs and drug classes. Identification of novel clinical opportunities (e.g., via drug combinations and repurposing).

## Supplementary Materials

The following are available online at https://www.mdpi.com/2227-9032/7/1/45/s1. Table S1: Selected MedDRA level 2 classes in FAERS. Table S2: MedDRA level 2 and 3 categories of the general MedDRA class ‘Social circumstances’, Table S3: Top 20 non-physiological (disease, condition, side-effect) MedDRA level 3 reaction categories in FAERS. Supplementary_Data.xls contains reaction (MedDRA level 3) statistics and DDI-outcome analysis.
