# Supplementary material for: Adverse Event Circumstances and the Case of Drug Interactions
Source: Healthcare (Basel). 2019 Mar 19;7(1):45. doi: 10.3390/healthcare7010045 (PMC6473808; doi:10.3390/healthcare7010045)
Supplement: Supplementary file 1 [file healthcare-07-00045-s001.zip › healthcare-456417-supplementary.docx]

Supplementary Materials: Adverse Event Circumstances and the Case of Drug Interactions

Theodoros G. Soldatos and David B. Jackson *

**Table S1.** Selected MedDRA level 2 classes in FAERS. The following MedDRA level 2 categories have been omitted from this table as they are discussed more in detail under the MedDRA level 3 classes presented in Table S3: MedDRA level 2 category ‘Complications associated with device’ of the more general MedDRA 1 class ‘General disorders and administration site conditions’; MedDRA level 2 categories ‘Off label uses and intentional product misuses/use issues’, ‘Overdoses and underdoses NEC’, ‘Procedural related injuries and complications NEC’, and ‘Exposures, chemical injuries and poisoning’ of the more general MedDRA 1 class ‘Injury, poisoning and procedural complications’; as well as categories ‘Device issues’, and ‘Product quality, supply, distribution, manufacturing and quality system issues’ of the MedDRA class ‘Product issues’.


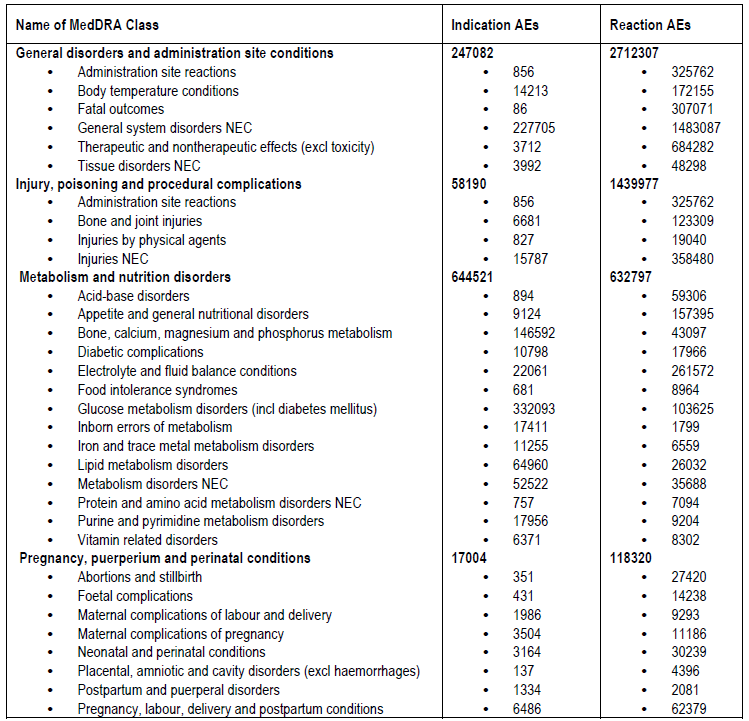


**Table S2.** MedDRA level 2 and 3 categories of the general MedDRA class ‘Social circumstances’. The general class ‘Social circumstances’ is linked to only 16268 as indication (0.24% FAERS) but to 103877 AEs as reaction (1.53% FAERS).


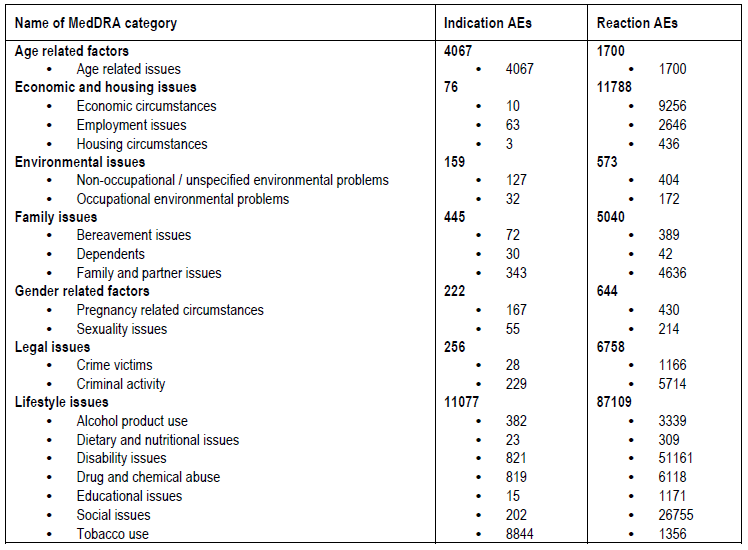


**Table S3.** Top 20 non-physiological (disease, condition, side-effect) MedDRA level 3 reaction categories in FAERS. The table lists selected MedDRA level 3 terms reported in more than 1000 AEs as reactions, and respective representative MedDRA level 4 subclasses for each category that are reported in more than 500 AEs. Most of these categories recapitulate and reflect classes omitted from Table S1.


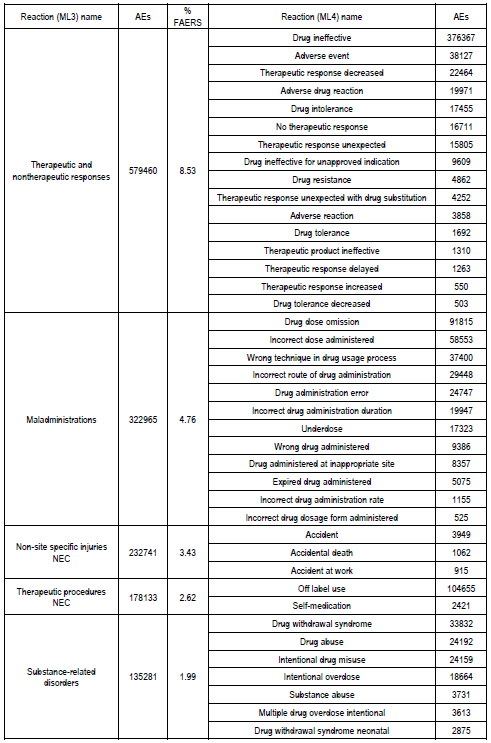


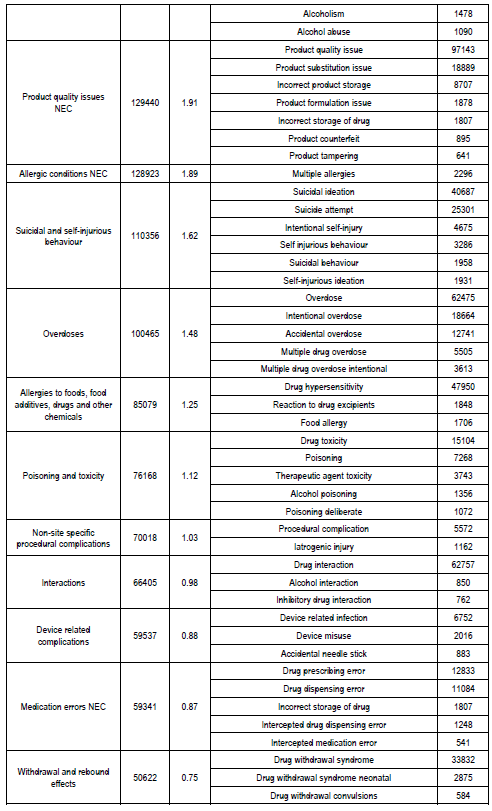


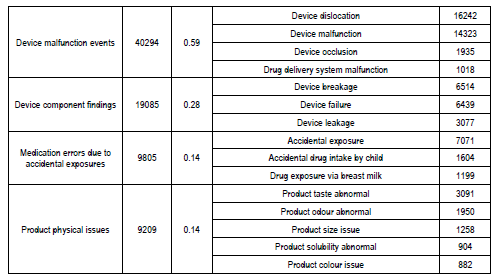


Name of MedDRA level 3 Reaction and Summary of DDI analysis results were summarized in a separated table


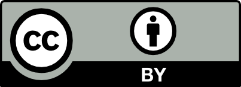
© 2019 by the authors. Licensee MDPI, Basel, Switzerland. This article is an open access article distributed under the terms and conditions of the Creative Commons Attribution (CC BY) license (http://creativecommons.org/licenses/by/4.0/).
